# Taurine Bromamine: Reactivity of an Endogenous and Exogenous Anti-Inflammatory and Antimicrobial Amino Acid Derivative

**DOI:** 10.3390/biom6020023

**Published:** 2016-04-21

**Authors:** Luiza De Carvalho Bertozo, Nelson Henrique Morgon, Aguinaldo Robinson De Souza, Valdecir Farias Ximenes

**Affiliations:** 1 Department of Chemistry, Faculty of Sciences, São Paulo State University (UNESP), Bauru 17033-360, Brazil; luiza_bertozo@yahoo.com.br (L.D.C.B.); arobinso@fc.unesp.br (A.R.D.S.); 2 Department of Chemistry, Institute of Chemistry, Campinas State University (UNICAMP), Campinas 13083-861, Brazil; nhmorgon@gmail.commailto

**Keywords:** taurine bromamine, tryptophan, melatonin, serotonin, singlet oxygen, nucleosides

## Abstract

Taurine bromamine (Tau-NHBr) is produced by the reaction between hypobromous acid (HOBr) and the amino acid taurine. There are increasing number of applications of Tau-NHBr as an anti-inflammatory and microbicidal drug for topical usage. Here, we performed a comprehensive study of the chemical reactivity of Tau-NHBr with endogenous and non-endogenous compounds. Tau-NHBr reactivity was compared with HOBr, hypochlorous acid (HOCl) and taurine chloramine (Tau-NHCl). The second-order rate constants (*k*_2_) for the reactions between Tau-NHBr and tryptophan (7.7 × 10^2^ M^−1^s^−1^), melatonin (7.3 × 10^3^ M^−1^s^−1^), serotonin (2.9 × 10^3^ M^−1^s^−1^), dansylglycine (9.5 × 10^1^ M^−1^s^−1^), tetramethylbenzidine (6.4 × 10^2^ M^−1^s^−1^) and H_2_O_2_ (3.9 × M^−1^s^−1^) were obtained. Tau-NHBr demonstrated the following selectivity regarding its reactivity with free amino acids: tryptophan > cysteine ~ methionine > tyrosine. The reactivity of Tau-NHBr was strongly affected by the pH of the medium (for instance with dansylglycine: pH 5.0, 1.1 × 10^4^ M^−1^s^−1^, pH 7.0, 9.5 × 10 M^−1^s^−1^ and pH 9.0, 1.7 × 10 M^−1^s^−1^), a property that is related to the formation of the dibromamine form at acidic pH (Tau-NBr_2_). The formation of singlet oxygen was observed in the reaction between Tau-NHBr and H_2_O_2_. Tau-NHBr was also able to react with linoleic acid, but with low efficiency compared with HOBr and HOCl. Compared with HOBr, Tau-NHBr was not able to react with nucleosides. In conclusion, the following reactivity sequence was established: HOBr > HOCl > Tau-NHBr > Tau-NHCl. These findings can be very helpful for researchers interested in biological applications of taurine haloamines.

## 1. Introduction

Hypochlorous acid (HOCl) and hypobromous acid (HOBr) are microbicidal agents produced when the white blood cells, neutrophils and eosinophils, respectively, are challenged by stimuli like bacteria and fungi [1]. Eosinophils, in particular, are associated with the host defense against parasitic helminth infections and asthma exacerbation [2,3]. These halogenating and oxidizing agents also play an important role in tissue damage associated with chronic inflammatory diseases [4,5]. For instance, eosinophilia, a characteristic of asthmatic subjects [6] has been associated with an increased level of 3-bromotyrosine, a biomarker of the damaging effects of HOBr [7].

The formation of these oxidants is due to the large amount of the enzymes myeloperoxidase (MPO) and eosinophil peroxidase (EPO) in these cells, which promote the catalytic oxidation of chloride (Cl^−^) and bromide (Br^−^) to HOCl and HOBr, respectively [8,9]. The catalytic mechanism involves the transient production of the redox active compounds, named compound I (MPO-I and EPO-I), which are two electron oxidized compared with the native enzyme. An important difference between MPO-I and EPO-I is their standard reduction potential [10]. Thus, while MPO-I (1.16 V) is able to oxidize efficiently both Cl^−^ and Br^−^ [11]; EPO-I (1.09 V) shows a large preference for Br^−^ [12]. This chemical feature, the higher plasma level of Cl^−^ (~100 mM) compared with Br^−^ (~100 µM), and the higher oxidant capacity of HOCl (1.28 V) compared with HOBr (1.13 V) [13], could suggest that the biological effect of HOBr would be irrelevant compared with HOCl. However, this does not seem to be the case, since there is significant evidence that HOBr is more reactive with several biomolecules compared to HOCl [14,15]. This phenomenon is the consequence of the higher electrophilicity of HOBr compared to HOCl [16].

Taurine bromamine (Tau-NHBr) is a chemical produced by the reaction between HOBr and the non-essential amino acid taurine. Yazdanbakhsh *et al.* provided the first application of Tau-NHBr and proposed its endogenous formation [17]. These authors found that stimulated eosinophils could be a source of Tau-NHBr, since these cells may produce HOBr, and taurine is abundantly present in leukocytes. It is interesting to note that this finding took place just one year after the discovery that eosinophils could produce and release HOBr [18].

Currently, Tau-NHBr is used as an anti-inflammatory and a topical antimicrobial drug. Some examples of its applications are its use as a therapeutic agent for the treatment of acne vulgares [19,20]; treatment of biofilm-associated infections on dental surfaces caused by *Pseudomonas aeruginosa* [21,22]; microbicidal activity against *Escherichia coli* and *Staphylococcus aureus* at insensitive body regions with low organic matter [23]; inhibition of the production of inflammatory mediators, such as prostaglandin E2 (PGE2), nitric oxide (NO) and pro-inflammatory cytokines [24]; and inhibition of degradation of TNF-α-induced Nuclear Factor-kappaB activation in Jurkat cells and myeloid-committed eosinophils [25]. The equivalent chlorinated haloamine is taurine chloramine (Tau-NHCl), which is the reaction product of the interaction between taurine and HOCl. Tau-NHCl is produced by activated neutrophils and released at inflammatory sites, inhibiting the production of inflammatory mediators [26]. An important chemical feature of Tau-NHCl, which makes this compound so interesting and extensively studied, is its mild oxidant capacity compared to its precursor HOCl. Thus, Tau-NHCl is able to oxidize selectively sulfhydryl residues in proteins [27], and to act as an endogenous antioxidant [28]. These aspects might be involved in the signaling pathways susceptible to Tau-NHCl [29,30]. There are also a large number of applications for Tau-NHCl as a topical anti-inflammatory and anti-infective drug [31].

In contrast to Tau-NHCl, for which the chemical properties have been intensively studied [32], much less is known about Tau-NHBr. This was our motivation for undertaking this study. Here, the reactivity of Tau-NHBr was evaluated and compared with HOBr, HOCl and Tau-NHCl, using several endogenous and non-endogenous chemicals. Using fast kinetic techniques, it was possible to measure the bimolecular rate constants of these reactions. We believe that this chemical data will be helpful for those interested in the application of this interesting compound.

## 2. Results and Discussion

### 2.1. Preparation and Stability of Tau-NHBr

Tau-NHBr can be prepared by reacting HOBr with taurine (Equation 1). However, it exists in equilibrium with its dibromamine form (Tau-NBr_2_) and, as has been demonstrated by Thomas *et al.*, pure Tau-NHBr is only obtained using a large excess of taurine. In our studies, we usually prepare stock solutions of Tau-NHBr by reacting 5 mM HOBr with 500 mM taurine in 50 mM phosphate buffer, pH 7.0, *i.e.*, a 100-fold excess of taurine. This solution was very stable when stored in the refrigerator and lost less than 20% of its initial concentration after five weeks [33].

(1) $$\begin{array}{ccc} {\mathrm{HO}}_{3}{\mathrm{SCH}}_{2}{\mathrm{CH}}_{2}{\mathrm{NH}}_{2} & +\;\mathrm{HOBr}\to & {\mathrm{HO}}_{3}{\mathrm{SCH}}_{2}{\mathrm{CH}}_{2}\mathrm{NHBr}\\ \left(\mathrm{Taurine}\right) & & (\mathrm{Tau-NHBr}), \end{array}$$

### 2.2. Reactivity with Tryptophan

**Figure 1:**
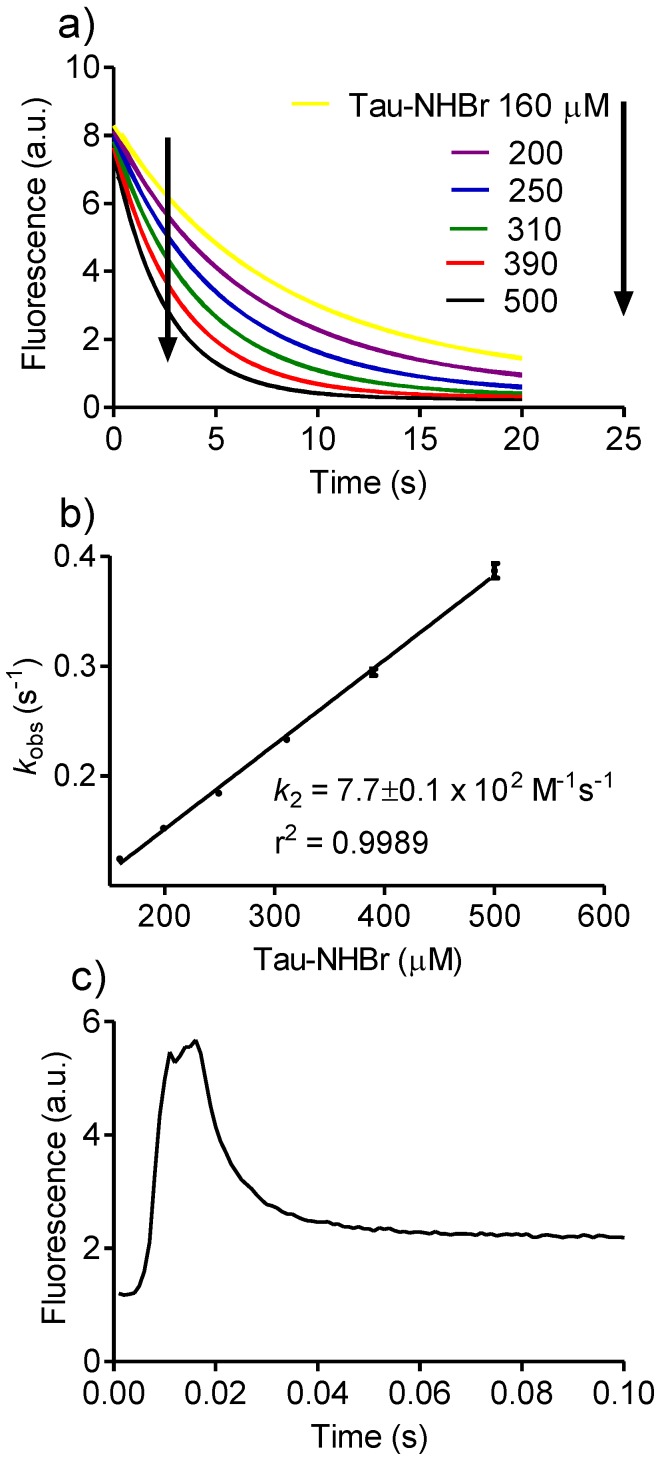
Reactivity of Tau-NHBr and HOBr with tryptophan. (**a**) Kinetic profile of tryptophan consumption for determination of *k_obs_* under pseudo-first-order experimental conditions. The reaction mixture was composed of 25 µM tryptophan and increasing concentrations of Tau-NHBr in 50 mM phosphate buffer, pH 7.0 at 25 °C. The stopped-flow system was set as follows: excitation, 280 nm LED; and emission, 325 nm cut-off filter; (**b**) Determination of the bimolecular rate constant (*k_2_*) for the reaction between tryptophan and Tau-NHBr; (**c**) Kinetic profile of tryptophan (25 µM) consumption by HOBr (50 µM). The results are the mean of three experiments.

Aiming to obtain a comprehensive knowledge of the chemical reactivity of Tau-NHBr, we used endogenous and non-endogenous compounds as potential targets. The first target was tryptophan, whose reactivity with HOCl and HOBr has been described [34,35]. Figure 1a shows the time-dependent fluorescence decay of tryptophan by the addition of Tau-NHBr. In these experiments, the concentration of tryptophan was kept constant at 25 µM and the concentration of Tau-NHBr was varied from 100 to 500 µM. From this pseudo-first-order experimental condition, the apparent second-order rate constant (*k*_2_) was calculated (7.7 × 10^2^ M^−1^s^−1^, Figure 1b). To gain insight into the significance of this value, it was compared with those obtained using HOBr, HOCl and Tau-NHCl. Figure 1a shows that, whereas 150 µM Tau-NHBr provoked the tryptophan fluorescence decay in about 20 s, HOBr caused the same effect in less than 0.02 s (Figure 1c). Unfortunately, this reaction was too fast to determine *k_2_*; however, its higher reactivity compared to Tau-NHBr was evident. Regarding HOCl, we took as reference a recent determination performed in our laboratory using exactly the same analytical protocol (*k_2_* = 8.1×10^4^ M^−1^s^−1^) [15]. Finally, and in agreement with the well-known low reactivity of Tau-NHCl [31], we found that this haloamine was unreactive with tryptophan. In fact, the experiments were done using up to a 10-fold excess of Tau-NHCl compared with Tau-NHBr, but no indication of consumption of tryptophan was observed for up to 10 min. From these results, the following reactivity sequence was obtained: HOBr > HOCl > Tau-NHBr > Tau-NHCl (unreactive).

**Figure 2:**
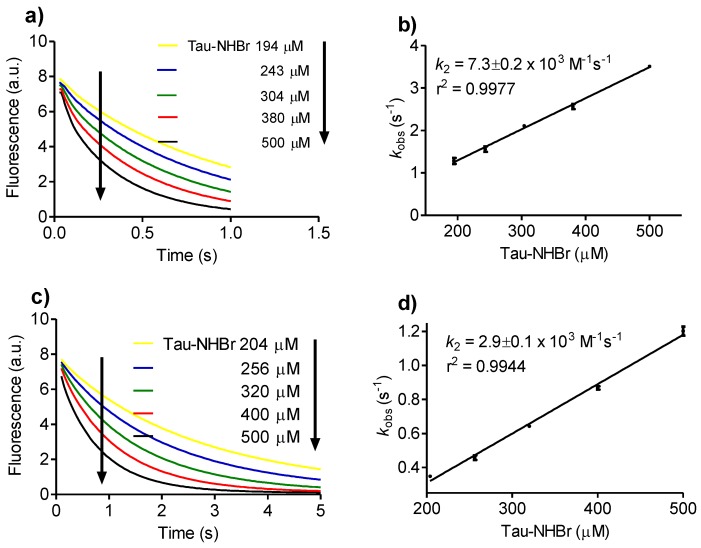
Reactivity of Tau-NHBr with melatonin and serotonin. Kinetic profile of (**a**) Melatonin and (**c**) Serotonin consumption for determination of *k_obs_* under pseudo-first-order experimental conditions. The reaction mixture was composed of 25 µM indoles and increasing concentrations of Tau-NHBr in 50 mM phosphate buffer, pH 7.0 at 25 °C. The stopped-flow system was set as follows: excitation, 280 nm LED; and emission, 325 nm cut-off filter. Determination of the bimolecular rate constant (*k_2_*) for the reaction between (**b**) Melatonin and (**d**) Serotonin with Tau-NHBr. The results are the mean of three experiments.

Besides tryptophan, the reactivity of Tau-NHBr with melatonin and serotonin was also obtained. These tryptophan derivatives have many physiological functions, including endogenous antioxidative activity [36,37]. Among others factors, this property is related to the lower one-electron reduction potential of these molecules compared to tryptophan (tryptophan E°’ = 1.01 V, melatonin E°’ = 0.95 V, and serotonin E°’ = 0.65 V) [38]. In agreement, the *k_2_* values obtained for melatonin (7.3 × 10^3^ M^−1^s^−1^) and serotonin (2.9 × 10^3^ M^−1^s^−1^) were about 10-fold higher compared to tryptophan (Figure 2).

### 2.3. Reactivity with Dansylglycine

In contrast to tryptophan, which has significant intrinsic fluorescence, other oxidizable amino acids, like methionine and cysteine, are not fluorescent. Tyrosine is also fluorescent, but with its maximum excitation/emission at 280/305 nm, our application of a stopped-flow system coupled to a LED source (280 nm) and the emission cut-off filters (305 nm) made the determination of *k_2_* unworkable. Thus, instead of a direct measurement of the reaction rate, an indirect procedure was performed by comparing the effect of these amino acids with the reaction rate of dansylglycine and Tau-NHBr. Dansylglycine is a fluorescent probe used for attribution of binding sites in albumin [39]. This probe was selected for several reasons, including our previous determination of its bimolecular rate constant with HOBr and HOCl [15], its fluorescence properties (λ_ex_ 360 nm, λ_em_ 550 nm), which made the spectral interference of the studied compounds minimal, and its application as a probe for tryptophan residues in albumin, as will be demonstrated below.

**Figure 3:**
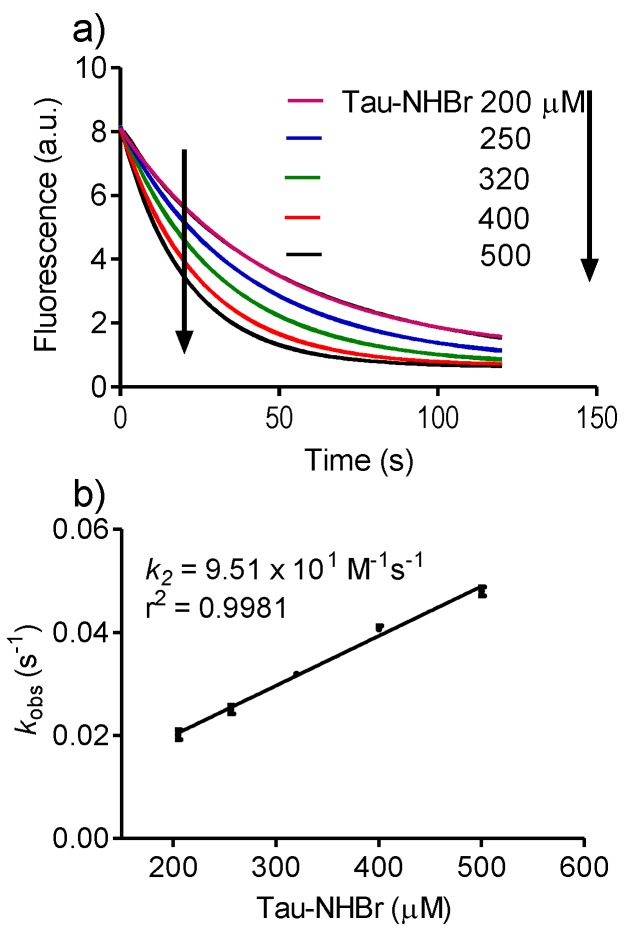
Reactivity of Tau-NHBr with dansylglycine. (**a**) Kinetic profile of dansylglycine consumption for determination of *k_obs_* under pseudo-first-order experimental conditions. The reaction mixture was composed of 25 µM dansylglycine (DG) and increasing concentrations of Tau-NHBr in 50 mM phosphate buffer, pH 7.0 at 25 °C. The stopped-flow system was set as follows: excitation, 360 nm LED; and emission, 475 nm cut-off filter; (**b**) Determination of *k_2_* for the reaction between dansylglycine and Tau-NHBr. The results are the mean of three experiments.

Before the use of dansylglycine for the comparison of the reactivity of these amino acids, the measurement of its apparent second-order rate constant with Tau-NHBr was performed (Figure 3). Corroborant with the tryptophan results, the obtained *k_2_* (9.5 × 10^1^ M^−1^s^−1^) was significantly lower than for HOBr (7.3 × 10^6^ M^−1^s^−1^) and HOCl (5.2 × 10^2^ M^−1^s^−1^) [15]. Tau-NHCl was totally unreactive with dansylglycine. The following reactivity sequence again was established: HOBr > HOCl > Tau-NHBr > Tau-NHCl.

### 2.4. Relative Reactivity with Tryptophan, Tyrosine, Cysteine and Methionine

Following the analytical protocol established above, the selectivity of Tau-NHBr with the oxidizable amino acids was evaluated by measuring and comparing tryptophan, tyrosine, cysteine and methionine in regard to their efficacy as inhibitors of the oxidation of dansylglycine.

**Figure 4:**
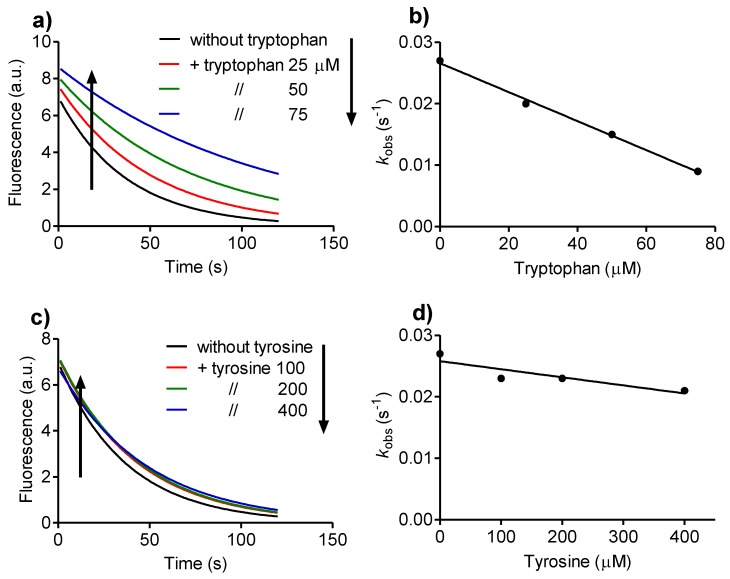
Tryptophan *versus* tyrosine as targets for Tau-NHBr. (**a**) Reaction of dansylglycine with Tau-NHBr and the effect of tryptophan; (**b**) Effect of tryptophan on the pseudo-first-order reaction rate of dansylglycine with Tau-NHBr; (**c**) Reaction of dansylglycine with Tau-NHBr and the effect of tyrosine; (**d**) Effect of tyrosine on the pseudo-first-order reaction rate of dansylglycine with Tau-NHBr. The reaction mixture was composed of 25 µM dansylglycine, 500 µM Tau-NHBr and increasing concentrations of amino acids in 50 mM phosphate buffer, pH 7.0. The stopped-flow system was set as follows: excitation, 360 nm LED; and emission, 475 nm cut-off filter.

The results displayed in Figure 4 show the effect of tryptophan and tyrosine in the fluorescence bleaching of dansylglycine. It can be observed that, whereas tryptophan was an effective competitor and significantly inhibited the depletion of dansylglycine, tyrosine was much less effective. From these experiments, the relative reactivity of the amino acids was calculated as the slope of the curve (*k_obs_* *versus* amino acid concentration). The results were tryptophan 2.4 × 10^−4^ ∆*k_obs_*/mM (Figure 4b) and tyrosine 1.3 × 10^−5^ ∆*k_obs_*/mM (Figure 4d), showing that tryptophan was about an 18-fold better inhibitor, or, in other words, 18-fold more reactive with Tau-NHBr compared with tyrosine.

**Figure 5:**
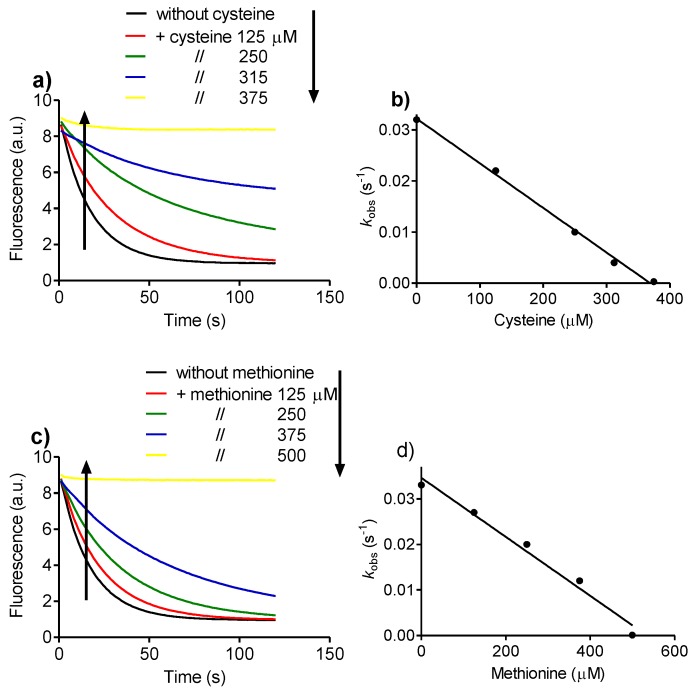
Cysteine *versus* methionine as targets for Tau-NHBr. (**a**) Reaction of dansylglycine with Tau-NHBr and the effect of cysteine; (**b**) Effect of cysteine on the pseudo-first-order reaction rate of dansylglycine with Tau-NHBr; (**c**) Reaction of dansylglycine with Tau-NHBr and the effect of methionine; (**d**) Effect of methionine on the pseudo-first-order reaction rate of dansylglycine with Tau-NHBr. The reaction mixture was composed of 25 µM dansylglycine, 500 µM Tau-NHBr and increasing concentrations of amino acids in 50 mM phosphate buffer, pH 7.0. The stopped-flow system was set as follows: excitation, 360 nm LED; and emission, 475 nm cut-off filter.

Following the same experimental concept, the effects of cysteine and methionine on dansylglycine bleaching were also measured. The slope of the curve of the pseudo first-order rate constant *versus* amino acid concentration was cysteine 8.7 × 10^−5^ ∆*k_obs_*/mM and methionine 6.5 × 10^−5^ ∆*k_obs_*/mM (Figure 5). Hence, the following sequence of relative reactivity of Tau-NHBr was established: tryptophan > cysteine ~ methionine > tyrosine.

### 2.5. Selectivity upon Tryptophan Residues in Proteins

As we have demonstrated above, Tau-NHBr has lower oxidant capacity compared to its precursor HOBr and HOCl. In this context, it is worth noting a principle of chemistry: lower reactivity usually implies greater selectivity. This principle seems to be applicable in our studies, because, in contrast to HOBr and HOCl, Tau-NHBr was able to oxidize tryptophan but not tyrosine. Following this idea, we suspect that our recent proposal for the selectivity of Tau-NHBr [40] and Tau-NBr_2_ [41] for tryptophan residues in albumin and lysozyme could be reinforced by these new findings. Thus, aiming to advance this proposal, dansylglycine was used to probe the depletion of tryptophan in human serum albumin (HSA).

Dansylglycine is a ligand of albumin. Its complexation can be monitored by an increased fluorescence quantum yield and a blue shift in the emission band, and, more importantly for our purpose, dansylated amino acids can be excited by fluorescence resonance energy transfer from tryptophan in HSA [42]. To make sure that this property is also applicable to dansylglycine, we added increasing concentrations of this compound to a fixed concentration of HSA. Our expectation was confirmed because dansylglycine was not fluorescent when excited at 295 nm; however, in the presence of HSA, the addition of increasing amounts of dansylglycine provoked a gradual quenching of the intrinsic fluorescence of the protein and a concomitant increase in the fluorescence of dansylglycine.

**Figure 6:**
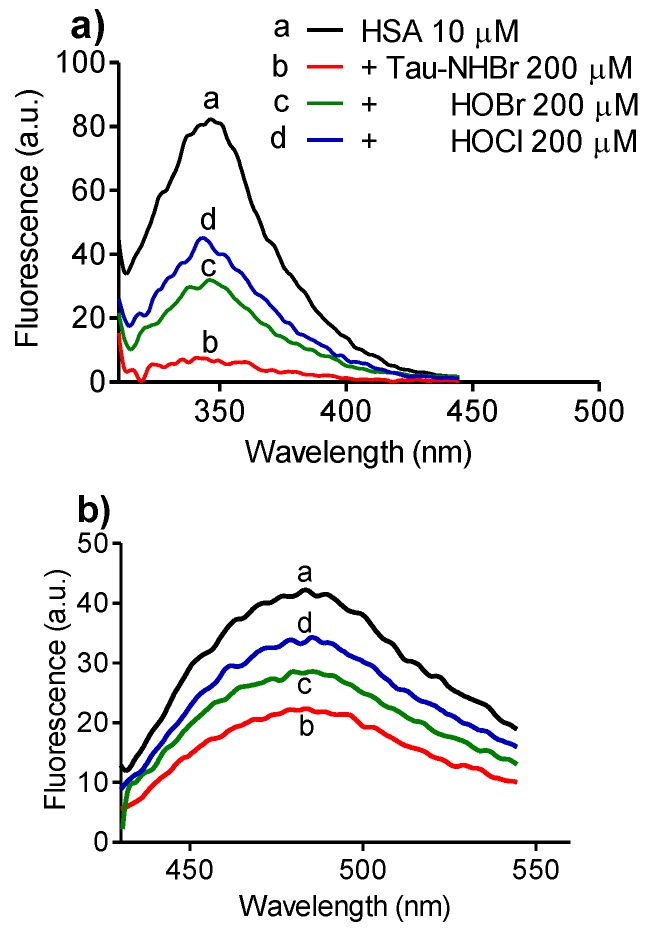
Oxidation of HSA by HOBr, HOCl and Tau-NHBr. (**a**) Reaction mixtures were composed of 10 µM HSA and 200 µM oxidants in 5.0 mM phosphate buffer, pH 7.0. After five minutes, 250 mM methionine was added and the HSA intrinsic fluorescence was measured at an excitation of 295 nm; (**b**) Addition of 5 µM dansylglycine to the oxidized protein and its fluorescence obtained by energy transfer from tryptophan residues.

Considering these findings, dansylglycine was used as a probe for evaluation of the consumption of tryptophan in HSA provoked oxidation. In these experiments, the oxidation was provoked by adding a 20-fold excess of the oxidants and, after five minutes, methionine was added to deplete the excess of oxidants. The results depicted in Figure 6a confirmed our expectation of selectivity because, though much less reactive with free tryptophan, Tau-NHBr was more effective than HOCl or HOBr in depleting the intrinsic fluorescence of HSA, which is mainly due to tryptophan residues when excited at 295 nm [43]. In others words, Tau-NHBr seems to act mainly on tryptophan residues of the protein. Figure 6b shows the effect of the addition of dansylglycine after oxidation and depletion of the oxidant excess. The band at 485 nm was lower in the sample oxidized with Tau-NHBr, which is an additional confirmation that tryptophan residues were more efficiently oxidized using this oxidant, since the measured fluorescence was due to energy transfer from the tryptophan residues in HSA.

### 2.6. Comparison between Tau-NHCl and Tau-NHBr

**Figure 7:**
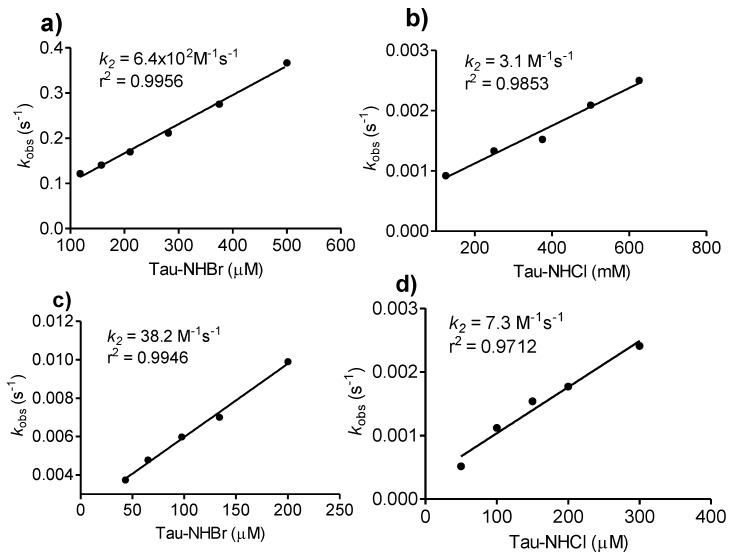
Tau-NHBr *versus* TauNHCl as oxidants of tetramethylbenzidine and curcumin. (**a,b**) Determination of bimolecular rate constant (*k_2_*) for the reaction between TMB and Tau-NHBr or Tau-NHCl. The reaction mixture was composed of 1.4 mM TMB and increasing concentrations of haloamines in 50 mM phosphate buffer, pH 7.0; (**c,d**) Determination of the bimolecular rate constants (*k_2_*) for the reactions between curcumin and Tau-NHBr or Tau-NHCl. The reaction mixture was composed of 10 µM curcumin and increasing concentrations of haloamines in 50 mM phosphate buffer, pH 7.0.

As we have demonstrated above, Tau-NHCl was unreactive with all studied compounds in this work; which is, indeed, in agreement with its well-established poor oxidant capacity [32]. Obviously, it does not mean that Tau-NHCl is completely devoid of oxidant capacity, as is confirmed, for instance, by its capacity to oxidize sulfhydryl residues in proteins [27]. Tau-NHCl is also able to oxidize 3,3′,5,5′-tetramethylbenzidine (TMB) in acid medium, the chromogenic substrate used for determination of the chlorination activity of MPO [44]. Thus, aiming to quantify the difference in reactivity between Tau-NHBr and Tau-NHCl, we used TMB as the target. It must also be noted that instead of an acidic pH, the reaction was conducted at pH 7.0, as was used for the other compound studies in this work. It is important to emphasize this point, because its reactivity is significantly lower at neutral pH, which is, indeed, the reason for the application of acidic medium for determination of Tau-NHCl activity using TMB [44]. The results depicted in Figure 7 confirmed our expectation, because, at pH 7.0, Tau-NHCl was able to oxidize TMB. Its oxidant efficacy was measured (*k_2_* = 3.1 M^−1^s^−1^) and, corroborant with the previous results, was about 100-fold lower than Tau-NHBr (*k_2_* = 6.4 × 10^2^ M^−1^s^−1^). The comparison was also made using the antioxidant curcumin, an oxidizable polyphenol, as the target. The rate constants were 7.3 M^−1^s^−1^ and 38.2 M^−1^s^−1^ for Tau-NHCl and Tau-NHBr, respectively (Figure 7).

### 2.7. pH Effect on Tau-NHBr Reactivity

**Figure 8:**
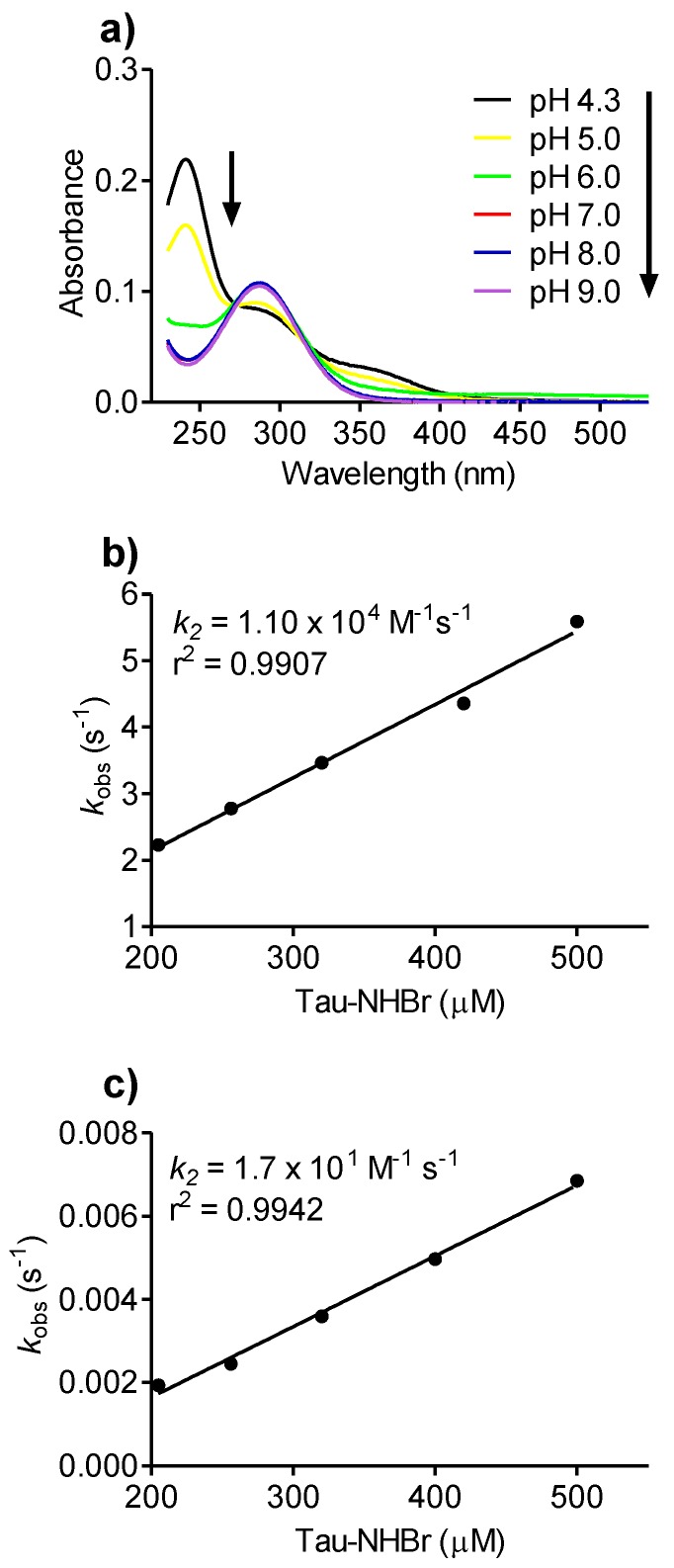
pH effect on Tau-NHBr reactivity. (**a**) Tau-NHBr (5 mM) was prepared in water using a 100-fold excess of taurine. The solution was diluted to 0.25 mM of NaH_2_PO_4_/Na_2_HPO_4_ (50 mM) solutions combined to reach the indicated pH; (**b,c**) Reactivity of Tau-NHBr with dansylglycine at pH 5.0 and 9.0.

In aqueous solutions, chloramine and bromamine are in equilibrium with their dichloramine and dibromamine forms [32,33]. These dihalogenated structures are the results of the disproportionation reaction, by which two molecules of Tau-NHX are converted to Tau-NX_2_ and taurine, respectively. The conversion of Tau-NHCl to Tau-NHCl_2_ is pH dependent, being favored at acidic pH [32]. Here, we found the same tendency for Tau-NHBr, which was progressively, converted to Tau-NBr_2_ as the pH decreased. The results depicted in Figure 8a show the absorbance increase at 241 and 346 nm and decrease at 288 nm, which provides evidence for the formation of Tau-NBr_2_ [33]. The chemical equation below shows the equilibrium between the mono- and dihalogenated forms as well as the pH dependence (Equation 2).

(2) $$\begin{array}{cccc} 2\;{\mathrm{HO}}_{3}{\mathrm{SCH}}_{2}{\mathrm{CH}}_{2}\mathrm{NHBr}\;+ & H^{+}↔ & {\mathrm{HO}}_{3}{\mathrm{SCH}}_{2}{\mathrm{CH}}_{2}{\mathrm{NBr}}_{2} & \;+{\mathrm{HO}}_{3}{\mathrm{SCH}}_{2}{\mathrm{CH}}_{2}{\mathrm{NH}}_{3}^{+}\\ \left(\mathrm{Tau-NHBr}\right) & & \left({\mathrm{Tau-NBr}}_{2}\right)\; & (\mathrm{Taurine}), \end{array}$$

Next, we studied the effect of pH on the reactivity of Tau-NHBr. Figure 8 shows the *k_2_* obtained for the reactions between dansylglycine and Tau-NHBr at pH 5.0 and 9.0. The rate constants were (pH 5.0) 1.1 × 10^4^ M^−1^s^−1^, (pH 7.0) 9.5 × 10^1^ M^−1^s^−1^ and (pH 9.0) 1.7 × 10^1^ M^−1^s^−1^. The pH dependence can be explained by taking into account the higher reactivity of the dihalogenated forms, as has been demonstrated for Tau-NCl_2_ [45].

### 2.8. Reactivity of Tau-NHBr with Hydrogen Peroxide and Formation of Singlet Oxygen

**Figure 9:**
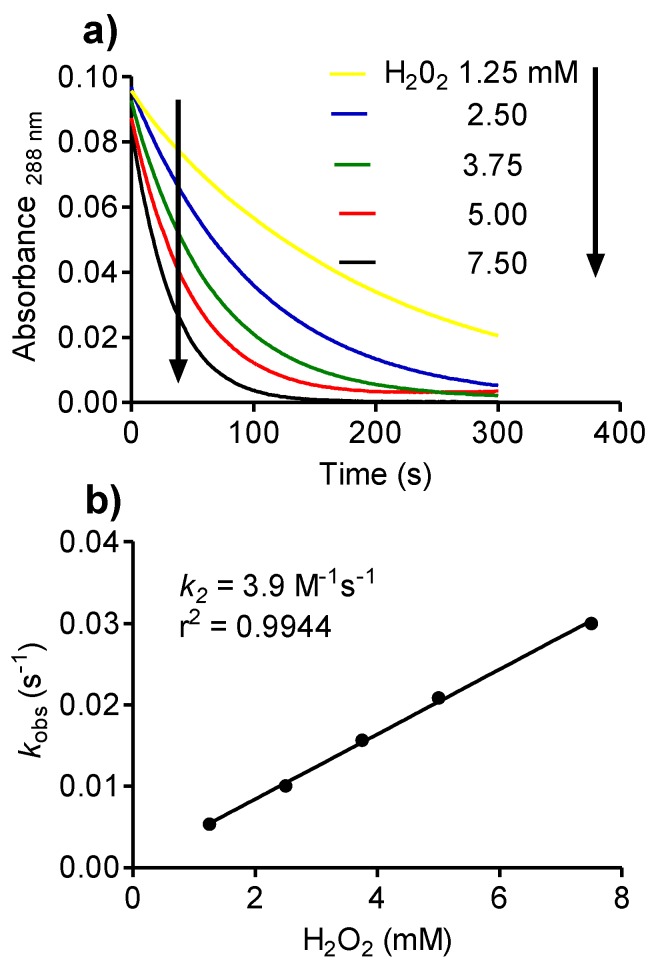
Reactivity of Tau-NHBr with H_2_O_2_. (**a**) Kinetic profile of Tau-NHBr consumption for determination of kobs under pseudo-first-order experimental conditions. The reaction mixture was composed of 250 µM Tau-NHBr and increasing concentrations of H_2_O_2_ in 50 mM phosphate buffer, pH 7.0; (**b**) Determination of the bimolecular rate constant (*k_2_*) for the reaction between H_2_O_2_ and Tau-NHBr.

Another property that distinguishes Tau-NHCl from Tau-NHBr is the capacity of the latter to react with H_2_O_2_ [33]. Here, this chemical property was confirmed and the *k_2_* (3.9 M^−1^s^−1^) obtained by measuring the decay of Tau-NHBr as a function of increasing concentrations of H_2_O_2_ (Figure 9).

**Figure 10:**
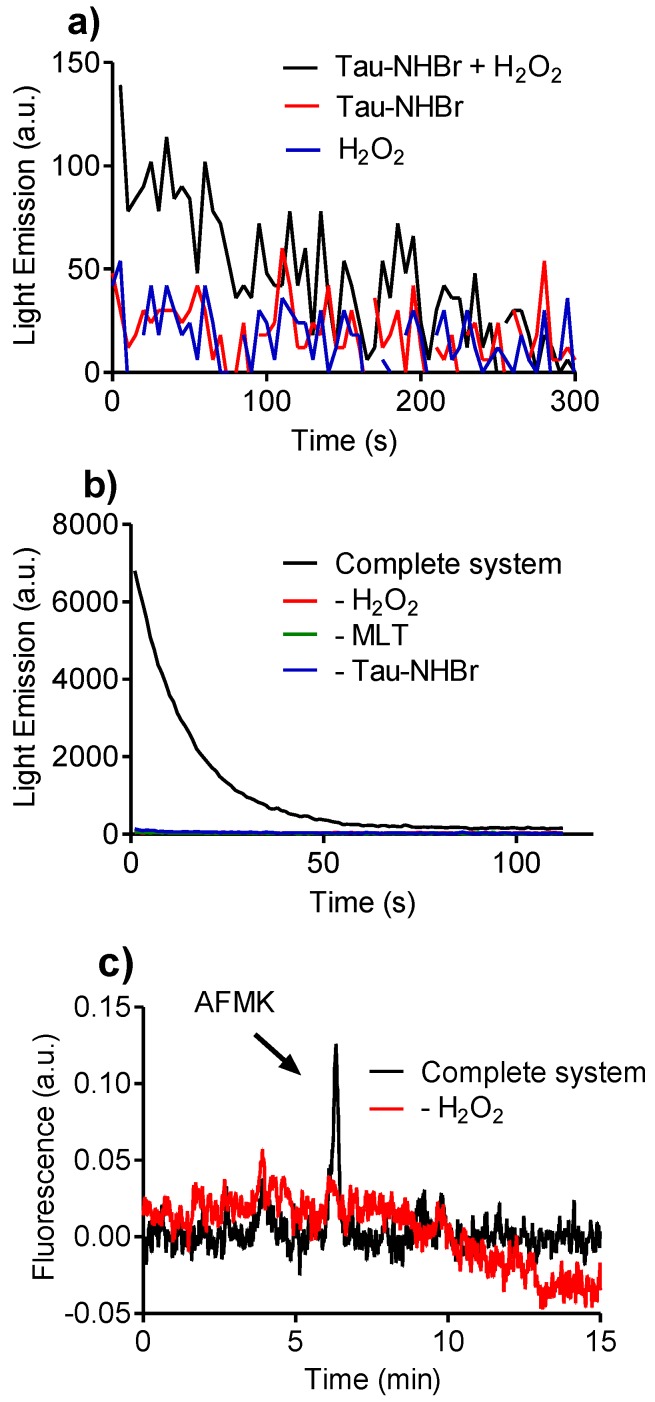
Evidence of oxygen singlet production. (**a**) Intrinsic light emission by the reaction of 0.25 mM Tau-NHBr with 5 mM H_2_O_2_ in 50 mM phosphate buffer, pH 7.0; (**b**) Melatonin-mediated light emission by the reaction consisting of 0.25 mM Tau-NHBr with 5 mM H_2_O_2_ and 1 mM melatonin in 50 mM phosphate buffer, pH 7.0 (complete system); (**c**) HPLC analysis of the reaction mixture and evidence of the formation of AFMK (peak at 6 min) obtained by comparison with a pure AFMK.

Activated eosinophils are an important *in vivo* source of singlet oxygen. The formation of this electronically excited form of oxygen is due to the reaction between HOBr and H_2_O_2_ [46]. We found that Tau-NHBr retains this capacity, as can be seen by the ultra-weak light emission produced during the reaction course originating from the decay of singlet oxygen (Figure 10a). It is important to emphasize that the extremely low light emission is due to the inadequacy of a conventional luminometer to measure the emission of singlet oxygen, which emits in the infrared region (1200 nm). Hence, to gain additional evidence of its production, we added melatonin to the reaction system. The results depicted in Figure 10b show that the light emission increased almost two orders of magnitude compared to the reaction without melatonin. The light emission is dependent on both H_2_O_2_ and melatonin, which excluded the possibility of direct interaction between melatonin and Tau-NHBr or melatonin and H_2_O_2_ as a source of the light emission. Thus, the increase in emission is consistent with the cleavage of the pyrrole ring of melatonin and formation of *N*-acetyl-*N*-formyl-5-methoxykynuramine (AFMK), a chemiluminescent reaction [47] that has been demonstrated between melatonin and singlet oxygen [48]. In accordance with this proposal, the formation of AFMK was also obtained here (Figure 10c).

### 2.9. Reactivity of Tau-NHBr with Linoleic Acid

**Figure 11:**
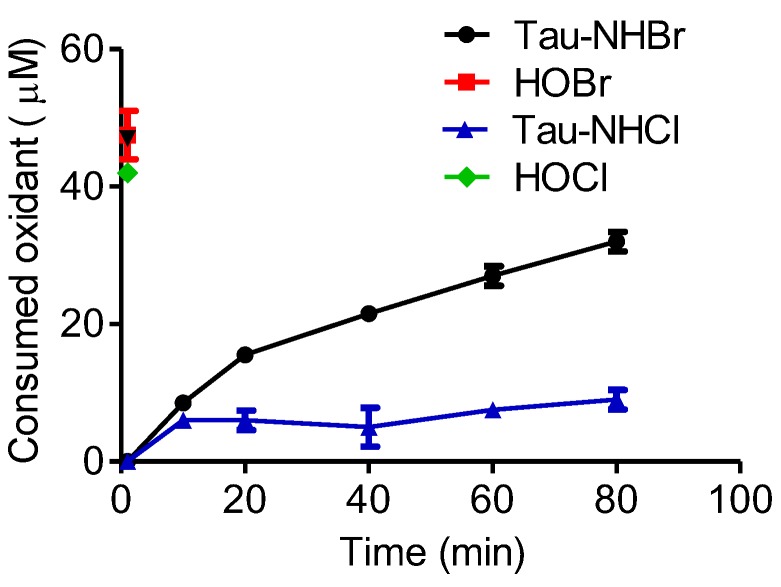
Reactivity with linoleic acid. The reaction mixtures were composed of 50 µM Tau-NHBr, Tau-NHCl, HOBr, or HOCl and 100 µM linoleic acid in 50 mM phosphate buffer, pH 7.0. After the indicated time, the remaining oxidants were measured by the TNB method. The results are the mean of three experiments. HOCl and HOBr were totally consumed in the first minute of reaction.

Among the biomolecules susceptible to the deleterious effects provoked by reactive oxygen species (ROS), unsaturated fatty acids occupy an important position. As electrophilic reagents, HOCl and HOBr are well established as inducers of the formation of halohydrin by reacting with the double bonds in mono- and polyunsaturated fatty acids [49,50]. For this reason, we also investigated the reactivity of Tau-NHBr with linoleic acid as a model of polyunsaturated fatty acids. In these experiments, linoleic acid was incubated with the oxidants and their remaining concentration was measured using the sulfhydryl reagent 5′-thio-2-nitrobenzoic acid (TNB). The results depicted in Figure 11 show that, while HOCl and HOBr reacted promptly with linoleic acid and were totally consumed just after one minute of incubation, Tau-NHBr reacted slowly, with only 50% consumed in 60 min of incubation. As could be expected from the previous results, Tau-NHCl was still less reactive.

### 2.10. Reactivity of Tau-NHBr with Nucleosides

**Figure 12:**
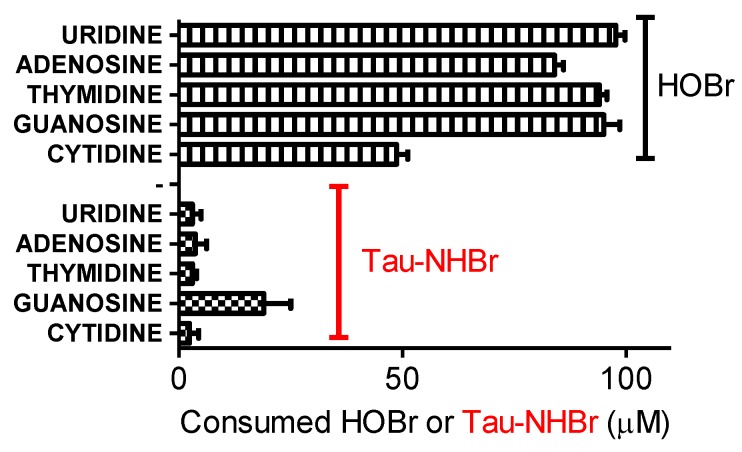
Reactivity of Tau-NHBr and HOBr with nucleosides. The reaction mixtures were composed of 100 µM Tau-NHBr or HOBr and 100 µM nucleosides in 50 mM phosphate buffer, pH 7.0. After 30 min the remaining oxidants were measured by the TNB method. The results are the mean of three experiments.

Other well established endogenous targets for hypohalous acids are nucleosides, nucleotides, DNA and RNA. For instance, 5-chlorouracil and 5-bromouracil are formed from the MPO catalyzed oxidation of uracil, and 5-chloro-2′-deoxycytidine, 8-chloro-2′-deoxyadenosine and 8-chloro-2′-deoxyguanosine result from the treatment of DNA with HOCl [51]. Recently, the miscoding properties of 8-chloro-2′-deoxyguanosine have been demonstrated, which highlights the potential importance of HOCl-mediated reactions in the pathogenesis of inflammation-driven carcinogenesis [52]. Similarly, the presence of 8-bromo-2′-deoxyguanosine has been proposed as a substance that may increase mutagenic potential at the site of inflammation [53]. For this reason, we also evaluated the reactivity of Tau-NHBr with nucleosides. However, the superposition of absorption spectra between Tau-NHBr, and the nucleosides impeded the direct measurement of their consumption. Hence, we again employed an indirect experimental approach by incubating the nucleosides with the oxidant and then measuring the remaining oxidant with the TNB reagent. The results depicted in Figure 12 show that, in contrast to HOBr, Tau-NHBr was significantly less reactive with nucleosides.

## 3. Experimental Section

### 3.1. Chemicals and Solutions

Taurine, tryptophan, tyrosine, methionine, cysteine, melatonin, serotonin, *N*-acetyl-*N*-formyl-5-methoxykynuramine (AFMK), TMB, 5,5′-dithiobis-(2-nitrobenzoic acid) (DTNB), dansylglycine, linoleic acid, salicylic acid, curcumin, adenosine, cytidine, guanosine, thymidine, uridine and HSA were purchased from Sigma-Aldrich Chemical Co. (St. Louis, MO, USA). The chemicals used for preparation of phosphate buffers and others solutions were of analytical grade. HOCl was prepared by diluting a 5% stock solution and the concentration was determined spectrophotometrically after dilution in 0.01 M NaOH, pH 12 (ε_292nm_ = 350 M^−1^cm^−1^). HOBr was synthesized by combining 100 mM HOCl and 200 mM NaBr in water [33]. Tau-NHCl was prepared by the addition of 5 mM HOCl to 50 mM taurine in 50 mM sodium phosphate buffer at pH 7.0 and its concentration was determined spectrophotometrically (ε_252nm_ = 429 M^−1^cm^−1^) [25]. Tau-NHBr was prepared by the addition of 5 mM HOBr to 500 mM taurine in 50 mM sodium phosphate buffer at pH 7.0 and its concentration was determined spectrophotometrically (ε_288nm_ = 430 M^−1^cm^−1^) [25]. TNB solution was prepared by dissolving 2 mM DTNB in 50 mM phosphate buffer, pH 7.0. Then, this solution was titrated to pH 12 with 0.1 M NaOH to promote its hydrolysis, and after 5 min the pH was brought back to 7.4 with hydrochloric acid. Then, its concentration was determined by its absorbance (ε_412nm_ = 14,100 M^−1^cm^−1^) [54]. A Perkin Elmer Lambda 35 UV-visible spectrophotometer (Manufacturer, Shelton, CT, USA) was used for the UV-Vis measurements.

### 3.2. Determination of Rate Constants

The reactivity of Tau-NHBr, HOBr and HOCl with the target molecules was obtained by comparing their bimolecular rate constants, which were obtained using pseudo-first-order experimental conditions. The fast-kinetic experiments were performed using a single-mixing stopped-flow system equipped with a high intensity LED source and cut-off filters (SX20/LED Stopped-Flow System, Applied Photophysics, City, UK). The observed pseudo-first-order rate constant (*k_obs_*) was obtained by fitting the fluorescence or absorbance decay of the studied compound to a single exponential decay equation, as follows (Equation 3):

(3) $$S\;=\;S_{0}\times e^{-k_{\mathrm{obs}}\;\times \;t},$$

where:

S is the fluorescence or absorbance as a function of time
S_0_ is the initial fluorescence or absorbance

From the *k*_obs_ values obtained at various concentrations, the bimolecular rate constants (*k_2_*) were calculated from the slope of the linear regression as follows (Equation 4):

*reaction rate* = *k*_2_ × [*A*] × [*B*], (4)

where [A] is the concentration of the haloamines or hypohalous acids and [B] is the concentration of the studied compounds. From the pseudo-first-order experimental condition, the apparent second-order rate constant (*k_2_*) was calculated (Equation 5).

*If* [*A*] ≫ [*B*], *then, reaction rate* = *k_obs_* × [*B*] *Then*, *k_obs_* = *k*_2_ × [*A*], (5)

The *k_2_* is the slope of the linear fit of *k_obs_ versus* [*A*]

The photophysical properties of the studied compounds and their intrinsic reactivity determined the experimental conditions used to monitor each reaction. The specific absorbance or excitation and emission wavelengths used for monitoring each reaction are shown in the figure legends. Unless otherwise stated, the concentrations of the target compounds were fixed at 25 µM, and the concentrations of the oxidants were in the range of 100 to 500 µM. The reactions were performed in 50 mM phosphate buffer, pH 7.0 at 25 °C.

### 3.3. Oxidation of Human Albumin and the Use of Dansylglycine as a Fluorescent Probe for Tryptophan Residues

The reaction mixtures were composed of 10 µM HSA and 200 µM oxidants in 50 mM phosphate buffer, pH 7.0. After five minutes, 250 µM methionine was added and the fluorescence was measured at an excitation wavelength of 295 nm and emission in the range of 310–450 nm was recorded. When present, dansylglycine was added at 5 µM and emission was measured in the range of 450–600 nm. The experiments were performed using a LS55 spectrofluorometer (Perkin-Elmer, Waltham, MA, USA). For the experiments where HSA was oxidized in the presence of dansylglycine, the stopped-flow system was set as follows: excitation, 280 nm LED, and emission, 455 nm cut-off filter.

### 3.4. Reactions with 3,3′,5,5′-tetramethylbenzidine (TMB), Curcumin, Hydrogen Peroxide and Linoleic Acid

3,3′,5,5′-tetramethylbenzidine: The reaction medium was composed of 1.4 mM TMB and increasing concentrations of haloamines in 50 mM phosphate buffer, pH 7.0. The reaction was monitored at 650 nm using conventional spectrophotometry.

Curcumin: The reaction medium was composed of 10 µM curcumin and increasing concentrations of haloamines in 50 mM phosphate buffer, pH 7.0. The reaction was monitored at 425 nm using conventional spectrophotometry.

Hydrogen peroxide: The reaction medium was composed of 250 µM Tau-NHBr and increasing concentrations of H_2_O_2_ in 50 mM phosphate buffer, pH 7.0. The reaction was monitored at 288 nm using conventional spectrophotometry. The reaction also was studied by chemiluminescence. In this case, the measurement of light emission was performed using a Centro LB 960 microplate luminometer (Berthold Technologies, Oak Ridge, TN, USA). The isolation and identification of oxidation products of melatonin was performed by high performance liquid chromatography in line with a fluorescence detector set at 340/460 nm (Jasco, Easton, MD, USA). The analyses were carried out isocratically on a Luna C18 reverse-phase column (250 × 4.6 mm, 5 µm) using 70:30 aqueous formic acid 0.1%/acetonitrile (flow rate 1.0 mL/min) as the mobile phase. The identification of AFMK as a product of the reaction was performed by comparison with a pure standard of this compound. 

Linoleic acid: The oxidants (50 µM) were incubated with 100 µM linoleic acid in 50 mM phosphate buffer, pH 7.0 for increasing time intervals. Then, the remaining concentration of oxidants was measured by the addition of 50 µM TNB, and the absorbance was measured at 412 nm (ε_412nm_ = 14,100 M^−1^cm^−1^).

## 4. Conclusions

Tau-NHBr, an endogenous oxidant, has also been used as an anti-inflammatory compound and topical antimicrobial agent. Here, we have identified several chemical properties of this promising drug. The most important was its selectivity regarding tryptophan residues in proteins. By measuring the bimolecular rate constant with several biomolecules, the following reactivity sequence was established: HOBr > HOCl > Tau-NHBr > Tau-NHCl. The reactivity of Tau-NHBr was strongly affected by the pH of the medium, a property related to the formation of the dibromamine form at acidic pH (Tau-NBr2). The formation of singlet oxygen was observed in the reaction between Tau-NHBr and H2O2. In conclusion, these findings could be very helpful for researchers interested in biological applications of this taurine haloamine.
